# Numerical Investigation of Phononic Crystal Based Film Bulk Acoustic Wave Resonators

**DOI:** 10.3390/nano11102547

**Published:** 2021-09-28

**Authors:** Linhao Shi, Weipeng Xuan, Biao Zhang, Shurong Dong, Hao Jin, Jikui Luo

**Affiliations:** 1 Ministry of Education Key Lab. of RF Circuits and Systems, College of Electronics & Information, Hangzhou Dianzi University, Hangzhou 310018, China; slheduedu@hdu.edu.cn (L.S.); zhangbiao123@hdu.edu.cn (B.Z.); 2 ZJU-Hangzhou Global Scientific and Technological Innovation Center, Zhejiang University, Hangzhou 310027, China; dongshurong@zju.edu.cn (S.D.); hjin@zju.edu.cn (H.J.); 3 Zhejiang Provincial Key Laboratory of Advanced Microelectronic Intelligent Systems and Applications, College of Information Science & Electronic Engineering, Zhejiang University, Hangzhou 310027, China

**Keywords:** bulk acoustic resonators, phononic crystals, high reflectivity, finite element method

## Abstract

Film bulk acoustic resonator (FBAR)-based filters have attracted great attention because they can be used to build high-performance RF filters with low cost and small device size. Generally, FBARs employ the air cavity and Bragg mirror to confine the acoustic energy within the piezoelectric layer, so as to achieve high quality factors and low insertion loss. Here, two-dimensional (2D) phononic crystals (PhCs) are proposed to be the acoustic energy reflection layer for an FBAR (PhC-FBAR). Four kinds of PhC structures are investigated, and their bandgap diagrams and acoustic wave reflection coefficients are analyzed using the finite element method (FEM). Then, the PhCs are used as the acoustic wave reflectors at the bottom of the piezoelectric stack, with high reflectivity for elastic waves in the specific frequency range. The results show that the specific PhC possesses a wide bandgap, which enables the PhC-FBAR to work at a broad frequency range. Furthermore, the impedance spectra of PhC-FBARs are very smooth with few spurious modes, and the quality factors are close to those of traditional FBARs with air cavities, showing the application potential of the PhC-FBAR filters with wide bandwidth and high power capability.

## 1. Introduction

With the continuous advancement of informatization and the rapid development of mobile technology—from smart homes to autonomous vehicles—there is an ever-growing demand for advanced radio frequency (RF) communication systems [1,2]. The filter is an indispensable component of assorted RF systems; the performance of the filters determines the overall quality of the communication systems.

However, traditional radio frequency filters are not able to meet the requirements for future wireless communication systems, due to structural and performance inadequacies. The ceramic filters have high power capacity and low insertion loss, but their dimensions are too large to meet the requirements of miniaturization [3]. The size of surface acoustic wave (SAW) filters is small, in the range of hundreds of microns, but they have low power capacity, and their working frequency is relatively low for the forthcoming information era [4,5]. Compared with ceramic and SAW filters, the thin-film bulk acoustic resonator (FBAR) is a kind of high-frequency resonator that has the advantages of small size, low insertion loss, high Q-value, high out-of-band rejection, and high power capacity [6,7,8]; thus, it has received particular attention in recent years.

FBARs are primarily present in three structures: the back-trench type, air-bag, and solid Bragg reflector structures. The first two structures use air as the acoustic reflection medium; the air presents high acoustic impedance mismatch, and can reflect the acoustic waves to the piezoelectric stack efficiently, leading to an FBAR with a high Q factor. The third structure uses multiple layers of materials with high and low acoustic impedance to form the Bragg mirror. The acoustic waves can be reflected to form the resonator; this structure is also named the solidly mounted resonator (SMR) [9]. The significant advantages of SMRs are their higher mechanical strength and robustness, and higher power capacity compared to the other two types of FBAR. However, this structure has some shortfalls, such as its limited working frequency range, and the smaller electromechanical coefficients (*k_t_*^2^), resulting in narrower bandwidth. To design high-performance filters with wide bandwidth, micro-acoustic resonators with high quality factor (Q) and high *k_t_*^2^ are necessary. Consequently, the thickness of Bragg layers for individual SMRs on one substrate must be optimized so as to have different frequencies, thus making the fabrication process much more complicated [10,11,12]. Therefore, innovative structures need to be explored for developing high-performance SMRs with broad bandwidth and a high quality factor.

Phononic crystal (PhC) is an artificial functional structure, which was first introduced by Yablonovithch in 1987 [13,14], and has been intensively explored over the past three decades [15,16,17]. The basic characteristics of PhC are as follows: PhC has one or several energy bandgaps so that the energy (such as elastic waves or light waves) will be completely confined and reflected within the structures when the frequency is within the energy bandgaps [18,19,20], i.e., the energy has low transmittance and high reflectivity when its frequency is within the bandgaps [21]. The Bragg reflector of SMRs can be regarded as one-dimensional (1D) PhC, which has a relatively small bandgap. Recently, 2D piezoelectric PhCs have gained much attention, as they have richer bandgap properties than those of 1D PhCs, such as wide and multiple bandgaps, high reflection, localized state control, negative refraction, etc. [22]. Two-dimensional PhCs have been used to control elastic waves and light waves by many researchers, such as manipulation of the transmission and direction of the TE-polarized and TM-polarized lights, spatial light filtering, etc. [23,24]. Muhammad et al. proved that the use of the acoustic bandgap generated by 2D PhC can effectively reduce the loss of acoustic waves through the anchor on the substrate for the Lamb wave resonators with frequencies in the range of 10–200 MHz, and significantly improve the Q factor of the resonators as compared with the traditional resonators [25,26,27,28]. Benchabance et al. utilized the bandgap characteristics of PhC on piezoelectric materials to enhance the performance of surface acoustic wave (SAW) filters with an operating frequency of 100–300 MHz [29]. Wu demonstrated SAW devices on micro air/silicon PhC theoretically and experimentally [30], and later applied the PhC-based SAW devices for use as waveguides [31] and high-Q resonant cavities [32]. The PhC structures used in the high-frequency (GHz) range can be achieved by micro/nanofabrication technologies [33]. However, thus far, the research on PhCs for elastic wave applications has mostly focused on surface acoustic wave or Lamb wave resonators, and little work has been done for high-frequency bulk acoustic wave resonators based on longitudinal waves. Thus, it is attractive and potentially beneficial to find some proper designs of PhCs for the bulk acoustic wave devices.

This paper demonstrates the feasibility of using 2D PhC as the acoustic reflective layer for the FBAR devices. Four PhC structures are studied theoretically, and the corresponding film bulk acoustic resonators (PhC-FBARs) are designed and analyzed numerically using FEM software. The frequency response, quality factor, and effective electromechanical coupling coefficient of the PhC-FBARs are investigated. Their performances are compared with traditional FBARs based on air cavities and Bragg mirrors. Results show that a properly designed two-dimensional PhC can be used as the bottom acoustic reflector of an FBAR. Compared with the SMR using a Bragg reflector layer (1D PhC), the 2D PhC could provide a wider frequency band for acoustic reflection, which is particularly important for the coming 5G era, which requires a wider frequency band and high-speed communication.

## 2. Calculation Method of PhC for Bandgap and Acoustic Wave Reflection

**Figure 1:**
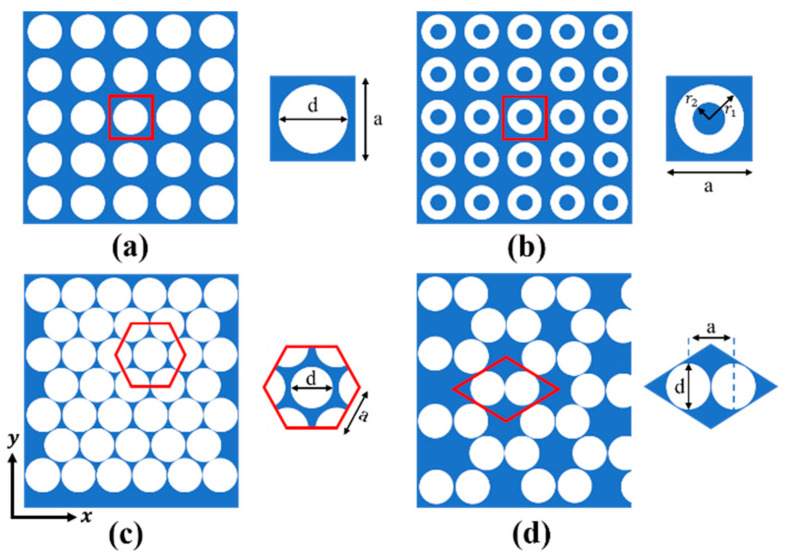
Phononic crystal structures of four different cell types: (**a**) square lattice, (**b**) square hollow cylinder lattice, (**c**) honeycomb lattice, (**d**) triangular lattice.

Four kinds of PhC structures, as shown in Figure 1, were explored to construct PhC-FBARs. The PhCs are composed of isotropic silicon and air, and the base material of the PhCs is silicon (the blue area), while the circular hole (or ring) is air (the white area). The parameters of the materials were as follows: Young’s modulus E=160 GPa, Poisson’s ratio u=0.265, and density $\rho =2330\;\mathrm{kg}/m^{3}$ for the isotropic silicon; and the acoustic wave velocity c=343 m/s for air. All four PhC structures use the same materials in the analysis.

The unit cells, as shown in Figure 1, were used to analyze the bandgap characteristics. To study the band structure of the phononic crystals, the finite element method was used for numerical analysis in this work. Based on the Bloch theorem, the Bloch periodic boundary condition was imposed on the structures. Due to the periodicity of phononic crystals, a representative unit cell can be applied to calculate the band structure of PhCs. For example, we assume that the coordinates of the unit cell are (*m*, *n*), and *a* is the lattice constant. Then, the Bloch condition states that when the elastic wave propagates in a 2D periodic medium, the displacement can be expressed as follows [34]:

(1) $${\tilde{u}}_{k}\left(x+ma,y+na\right)={\tilde{u}}_{k}\left(x,y\right)\exp\;\left(-i\left(k_{x}ma+k_{y}na\right)\right)\;$$

where  u is the incremental displacement, which represents the vibration amplitude of the particle, and $\;k_{x}$,$\;k_{y}$ are the components of the Bloch wave vector in the *x* and *y* directions, respectively, defined in the reciprocal lattice vector space.

It can be seen from Equation (1) that for a specific wave vector, *k*, there must be a corresponding wave vector k in the first Brillouin zone, and the waveforms of all particles are identical. The Brillouin zone is a region in space centered at the origin of the reciprocal lattice. Generally, the minimum incompressible region in the first Brillouin region of the lattice obtained by the symmetrical operation of the crystal point group is called the irreducible Brillouin region. Therefore, we only need to study the situation where the wave vector k falls within the first Brillouin zone.

**Figure 2:**
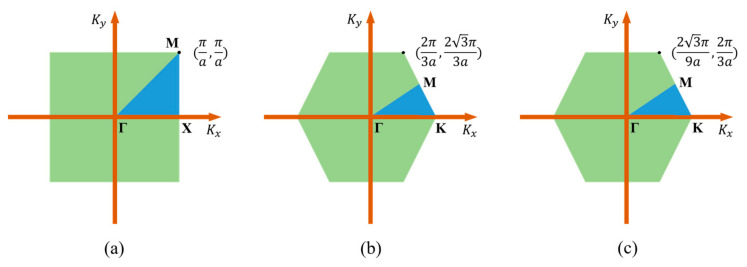
Three kinds of crystal lattices of the first Brillouin zone in reciprocal space for: (**a**) square lattice and square hollow cylinder lattice, (**b**) honeycomb lattice, (**c**) triangular lattice.

The FEM-based calculation was conducted by utilizing COMSOL Multiphysics 5.4. Based on the theoretical analysis above, we only need to draw the first Brillouin zone, calculate $k_{x}$ and $k_{y}$ of the wave vector, and finally apply the Floquet boundary conditions to the PhC. As discussed above, four PhCs with different structures were investigated. The first Brillouin zone corresponding to the square lattice and square hollow cylinder lattice can be treated identically, as shown in Figure 2a. The first Brillouin zones of the honeycomb and triangular lattices are shown in Figure 2b,c, respectively.

The displacement ratio between the output and input ports at different frequencies—i.e., the amplitude–frequency response—was utilized to calculate the acoustic wave transmission coefficient. Since PhC can prevent the propagation of elastic waves if their frequencies fall into the bandgap of the PhC, the amplitude–frequency response in the frequency range corresponding to the bandgap should be significantly less than the unity. The attenuation coefficient of the amplitude–frequency response curve can reflect the attenuation ability of the elastic wave propagation when the frequency is located in the gap range, which corresponds to the energy band diagram.

**Figure 3:**
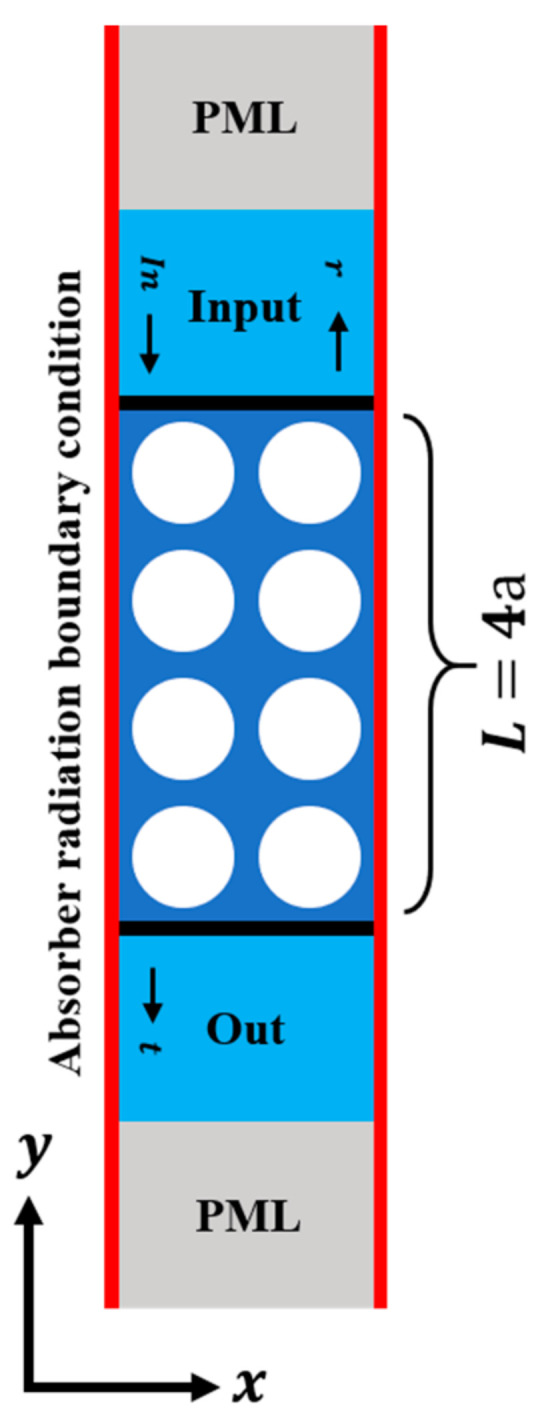
The schematic of a cell used for analysis of reflection coefficient characteristics.

The FEM method was also used to calculate the transmission characteristics (reflection coefficient) of the PhC with a finite period structure. Assume that the wave propagates in the y direction, as shown in Figure 3. Perfect matching layers (PMLs) are used on the boundary of the top and bottom sides to absorb any kind of elastic waves, with square lattice PhC as the example (the analysis method for other structures is the same). In the simulation, four layers of the PhC structure were used in the y direction to analyze the reflection coefficient. To simplify the analysis, we assumed that there are infinite periods in the x direction, so periodic boundaries were used at the left and right boundaries of the model. The solid black line on the top indicates the position where the acoustic excitation source is stimulated, while the solid black line at the bottom is the position of the measured output wave. In the mechanic’s module of COMSOL, we placed both the *y*-direction displacement (longitudinal excitation) and the *x*-direction displacement (lateral excitation) $P_{\mathrm{in}}$ on the input port. Then, the response $P_{\mathrm{out}}$ was obtained at the output port. The transmission loss and reflection coefficient are defined as:

(2) $$T=\frac{P_{\mathrm{out}}}{P_{\mathrm{in}}}$$

(3) R=1−T

## 3. Band Diagram and Acoustic Wave Reflection Analysis of the Four PhCs

Based on the theoretical analysis discussed in Section 2, the band diagram characteristics and reflection coefficients of the four different PhCs are discussed. It was found that there are multiple bandgaps in the PhCs; for convenience, the dependence of the first bandgap characteristic on the fill factor is analyzed in detail. The fill factor is defined as the area ratio of the air hole to the unit cell, which means that the lattice constant a is fixed while the diameter of the air hole is varied. The analyzed frequency range is from 0 GHz to 10 GHz, which covers the typical working frequency of FBAR devices planned for future communication systems.

**Figure 4:**
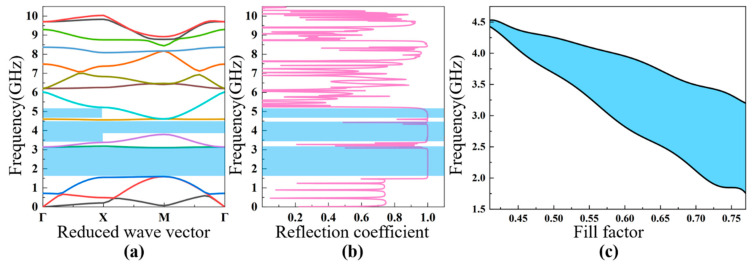
The calculated bandgap diagram (**a**) and reflection coefficient (**b**) of the square lattice PhC with the lattice parameters of $a=0.9\;\mu m,\;\frac{d}{2}=0.44\;\mu m$. The dependence of the width of the bandgap on the fill factor (**c**).

The calculated bands’ structure for the square lattice PhC (Figure 1a) is shown in Figure 4a. The lattice constant and the air hole diameter of this PhC are $a=0.9\;\mu m,\;\frac{d}{2}=0.44\;\mu m$, respectively. There are three obvious bandgaps in the frequency range from 0 to 10 GHz, including two full bandgaps and one Γ–X directional bandgap. The first bandgap ranges from 1.7 GHz to 2.9 GHz, with a gap width of 1.2 GHz; the second full gap is between 3.5 GHz to 4.4 GHz; and the directional bandgap is between 4.6 GHz and 5.1 GHz. The acoustic reflection coefficient is shown in Figure 4b, showing the relationship between the reflection coefficient and the energy band diagram. If propagation of the acoustic waves is within the bandgaps, then the reflection coefficient is close to the unity. In this case, the PhC can be utilized as the acoustic reflection layer for FBARs working in those frequency ranges. In the following part, we chose the first bandgap, from 1.7 GHz to 2.9 GHz, for a detailed investigation. Figure 4c illustrates the dependence of the first bandgap of the square lattice PhC on the fill factor, showing that the increase in the fill factor (increase in the air hole size) with a fixed lattice constant widens the bandgap of the low-frequency-range acoustic waves, which increases from 0.2 GHz at a fill factor of 0.42 to ~1.6 GHz at a fill factor of 0.8.

**Figure 5:**
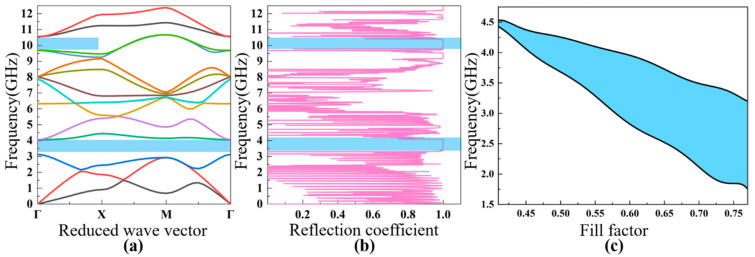
The calculated band diagram (**a**) and reflection coefficient (**b**) of the square hollow cylinder lattice PhC with the lattice parameter of a=0.9 μm, and the outer and inner radius of the cylinder $r_{1}=0.4\;\mu m\;\mathrm{and}\;r_{2}=0.3\;\mu m$, respectively. The dependence of the width of the bandgap on the fill factor (**c**).

Figure 1b shows the constitution of the second PhC with a square hollow cylinder structure, where the lattice constant is a=0.9 μm, and the outer and inner radius of the cylinder are $r_{1}=0.4\;\mu m\;\mathrm{and}\;\;r_{2}=0.3\;\mu m$ respectively. It can be observed in Figure 5a,b, in the frequency range between 0 to 11 GHz, that there are two bandgaps, with one full gap and the other a directional gap. In this PhC structure, it is noteworthy that the full frequency bandgap can be enlarged and reduced by changing the ratio of $r_{2}/r_{1}\;$ [35]. Similarly, we chose the first bandgap (3.3–4.0 GHz) for detailed investigation for FBARs. Figure 5c exhibits dependence of the first bandgap characteristic of this PhC on the fill factor, indicating that the increase in the fill factor (increase in the air hole size) leads to a wider low-frequency bandgap, similar to that of the square lattice PhC, as can be seen in Figure 4c and Figure 5c. This is because the two PhCs of square lattice and square hollow cylinder lattice have similar Brillouin zones.

**Figure 6:**
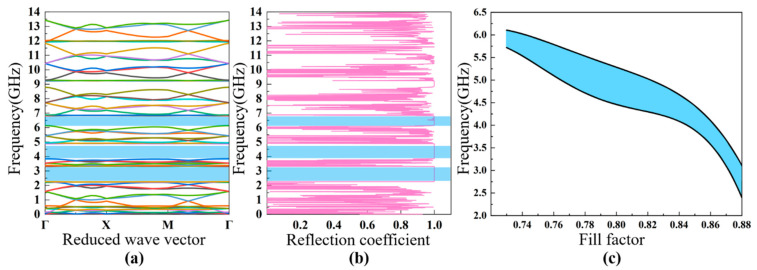
The calculated band diagram (**a**) and reflection coefficient (**b**) of the honeycomb lattice PhC with the lattice parameters of $a=0.9\;\mu m,\;\frac{d}{2}=0.44\;\mu m$. The dependence of the width of the bandgap on the fill factor (**c**).

Figure 1c shows the hexagon lattice PhC structure with elementary unit cells, which is derived from a two-dimensional triangle lattice model. A fill factor of 88% was used for this simulation, and the lattice constant and air hole diameter were $a=0.9\;\mu m,\;\frac{d}{2}=0.44\;\mu m$, respectively. From the bandgap characteristics shown in Figure 6a, there are three full frequency bandgaps—2.4–3.1 GHz, 4.0–4.6 GHz, and 6.3–6.6 GHz—and the bandwidths of all of the bandgaps are more than 0.5 GHz. Figure 6b shows the acoustic reflection coefficient of the PhC structure. Figure 6c shows the dependence of the first bandgap frequency range on the fill factor, which is relatively constant for the frequency range investigated, as compared to those of the square lattice PhCs discussed above.

**Figure 7:**
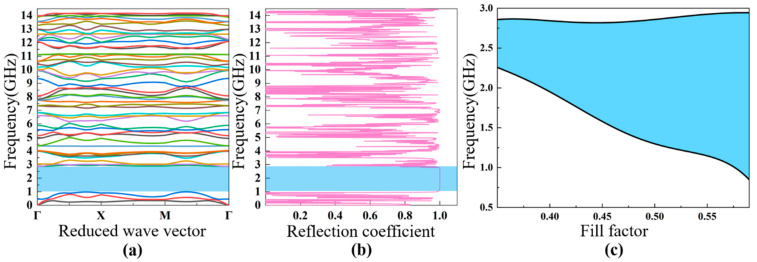
The calculated bandgap diagram (**a**) and reflection coefficient (**b**) of the triangular lattice PhC with the lattice parameters of a=0.9 μm, $\frac{d}{2}=0.43\;\mu m$. The dependence of the width of the bandgap on the fill factor (**c**).

The 2D PhC structure of Figure 1d is composed of a honeycomb array of air cylinders embedded in the silicon. The distance between two lattice cells is a=0.9 μm, and the air hole diameter is $\frac{d}{2}=0.43\;\mu m$. The bandgap characteristics and acoustic reflection coefficient of the PhC structure were calculated with the results shown in Figure 7a,b, respectively. It can be seen that there is only one full frequency bandgap in the range from 1.05 GHz to 2.9 GHz, with a bandwidth of 1.4 GHz. Figure 7c illustrates the dependence of the bandwidth of the bandgap on the fill factor, showing that the bandwidth increases from the initial ~1.4 GHz to ~3 GHz when the fill factor increases from 0.35 to 0.6. Based on the reflection ability and extreme broad bandgap, we used this as the bottom acoustic reflection layer for the analysis of FBAR devices.

## 4. Analysis and Discussion of PhC-FBARs

**Figure 8:**
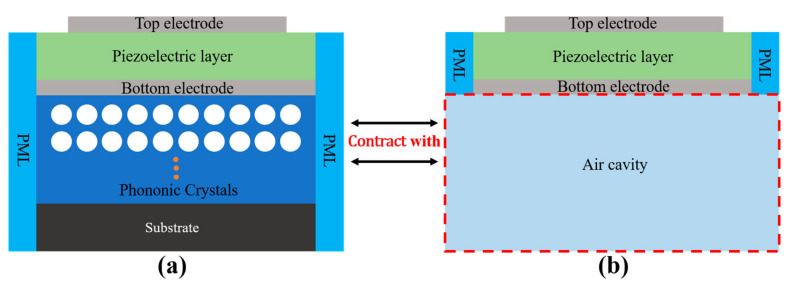
Two-dimensional structure of a bulk acoustic wave resonator with a PhC structure (**a**). Traditional FBAR with the air cavity, used for modeling (**b**).

The working principle of FBARs requires a total acoustic wave reflection boundary on the substrate [36]. We have proven that the acoustic reflection coefficients of the four PhC structures in the specific frequency bands are close to the unity; thus, they can be used as the acoustic reflection layer for the construction of wide-bandwidth FBARs. For the following section, the PhC-FBAR analysis was conducted based on the PhC structures discussed above. The air cavity in the FBAR configurations was substituted with one of the PhCs for calculation, and their results were compared with the corresponding FBARs with the air cavity. Figure 8a,b show the structure diagrams used for the simulations for the PhC-FBARs and traditional air cavity FBARs, respectively. For clarification, the FBAR structure with PhC as the reflective structure is called PhC-FBAR, while the traditional FBAR with an air cavity is called Air-FBAR.

From the top to the bottom of the PhC-FBAR, the structures have the following layers, in sequence: the top electrode, piezoelectric film, bottom electrode, PhC structure, and the substrate. The piezoelectric film AlN and metal Mo were used in this simulation. Based on the analysis of the bandgap characteristics of the four kinds of PhC, the resonant frequencies of the FBARs were designed to be within the bandgaps. The performance of the PhC-FBARs is compared with those of the Air-FBARs with the same piezoelectric stacks. From the PhC bandgaps calculated, we set the resonant frequencies of PhC-FBARs with the PhC structure of the square lattice, square hollow cylinder lattice, honeycomb lattice, and triangular lattice at 2.4 GHz, 3.4 GHz, 2.1 GHz, 2.4 GHz, respectively, so that the specific thicknesses of the AlN piezoelectric layers needed to be selected to match their frequency ranges. The corresponding thicknesses of the AlN layers were 1 μm, 0.6 μm, 1.2 μm, and 1 μm, respectively. The thicknesses of the other layers were kept the same—namely, 200 nm for the upper Mo electrode, and 250 nm for the lower electrode. All of the material parameters used in this simulation are summarized in Table 1.

The characteristics of the PhC-FBARs and the Air-FBARs were mainly assessed by quality factor Q and effective electromechanical coupling coefficient $K_{eff}$. In the analysis, the loss of all of the materials was not considered, except for the loss of the piezoelectric layer. The mechanical damping loss and dielectric loss of the AlN piezoelectric material were fixed at 0.001 and 0.01, respectively. The Q factor represents the ratio of the stored energy to the energy consumed in the FBAR. The formula to find the Q factor is as follows:

(4) $$Q\left(f\right)=\frac{f}{2}*\;\left|\frac{\partial Z}{\partial f}\right|,\;K_{eff}=\frac{\pi^{2}}{4}*\left(\frac{f_{a}-f_{r}}{f_{a}}\right)$$

where $f_{r},\;f_{a}\;$ represent the resonance and anti-resonance frequencies, respectively, while Z is the impedance of the device, obtained from the two electrodes of the FBAR device.

**Figure 9:**
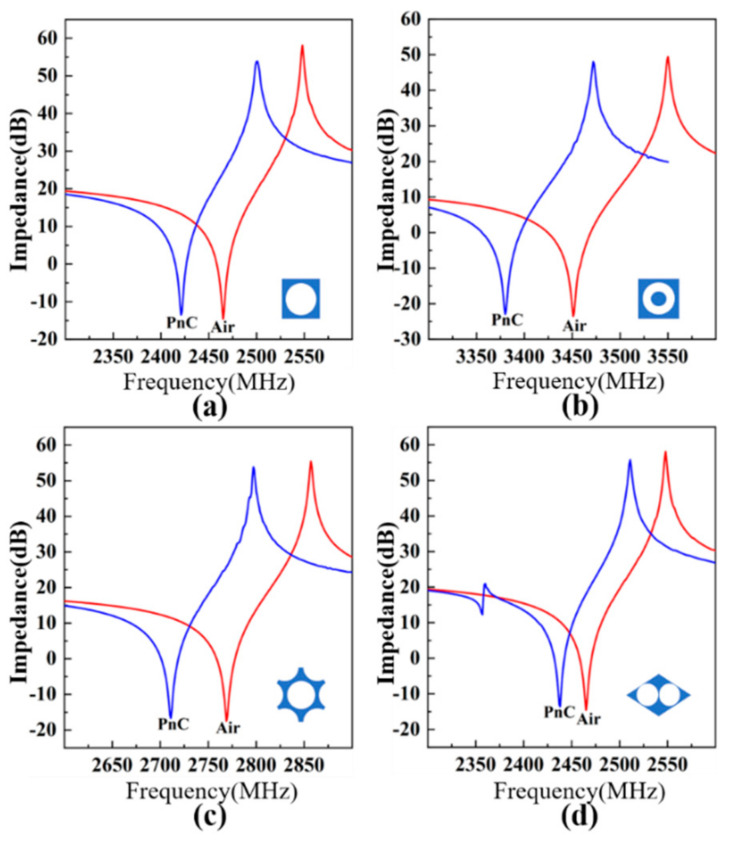
Comparison of impedance curves of the PhC-FBARs and Air-FBARs with the same piezoelectric stacks for the PhC structures of (**a**) square lattice, (**b**) square hollow cylinder lattice, (**c**) honeycomb lattice, and (**d**) triangular lattice.

The impedance spectra of the four PhC-FBARs and the corresponding Air-FBARs are shown in Figure 9. 

It can be seen that each of the resonance frequencies of the PhC-FBARs is slightly lower than that of the corresponding Air-FBAR. For instance, the resonance frequency of the first PhC structure is 2421.3 MHz, while the frequency for the corresponding Air-FBAR is 2465.1 MHz. The main reason for this phenomenon is the mass loading effect from the PhC structures as compared to air (assume it has no mass) as the reflection layers. The summaries and comparisons of four PhC-FBARs and Air-FBARs are summarized in Table 2. The quality factor Q of the PhC-FBARs is a little lower than that of the corresponding Air-FBARs. Based on the above analysis, we know that the reflection coefficient of the four PhCs is close to that of air, but never more than that of air. This means that a tiny number of acoustic waves will still leak into the substrate. Hence, they will have lower Q-values compared to those of Air-FBARs. For the FBAR resonator, there are several elements that will affect the Q factor, such as the Ohmic loss and acoustic loss induced by the selection of electrode material [37,38], the spurious wave mode that leads to energy loss [39,40], and the effect of the electrode shape [41]. The detailed analysis of the Q factor is beyond the scope of this work. In this work, we only consider the mechanical damping loss and dielectric loss of the AlN piezoelectric material.

**Figure 10:**
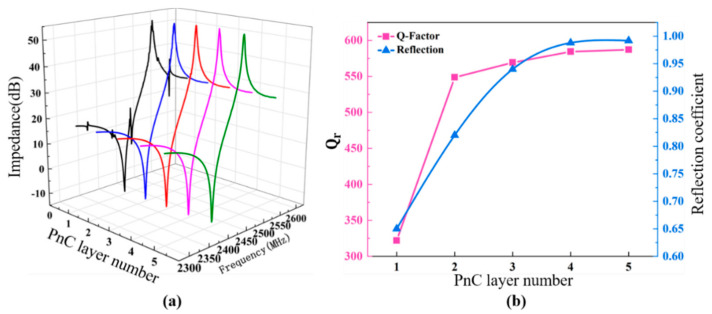
Impedance spectrum of the PhC-FBAR based on a triangular lattice PhC with different numbers of PhC layers (**a**), and the corresponding acoustic reflection coefficient of the PhC and Q_r_ of the PhC-FBAR at the resonance frequency (**b**).

The relationship between the Q factor of the PhC-FBARs and the number of periodic longitudinal rows (we may call this the PhC layer) is further analyzed. Although PhCs are able to reflect most of the acoustic energy, a small portion of it will still leak into the substrate, reducing the performance of the FBARs. Therefore, it is necessary to ensure that the PhC has a high reflectivity in the bandgap. Generally speaking, as the number of PhC layers increases, the value of the reflection coefficient will increase, and finally approach a stable value [42]. The calculated result is shown in Figure 10a for a square lattice PhC. There are some spurious resonances when the number of PhC layers is less than four, and the impedance spectra become smoother with no spurious resonance when more than four PhC layers are used. The reflection coefficient and the quality factor Q show similar tendencies concerning the increased number of PhC layers. It can also be noted that the Q factor improves significantly when the PhC layer increases from one to two layers, and it becomes much smaller when the number of PhC layers increases further; thus, it can be concluded that four PhC layers are necessary to achieve FBARs with good performance.

**Figure 11:**
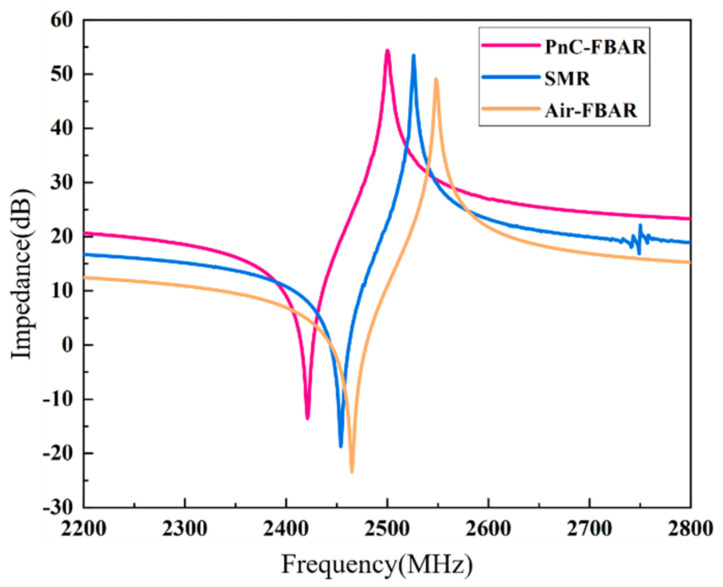
Comparison of impedance spectra of three kinds of acoustic isolation structures with the same piezoelectric stack.

As mentioned above, the Bragg reflector layer of SMRs is also a one-dimensional PhC structure, and it is necessary to compare the performance of the PhC-FBARs with that of SMRs. Figure 11 shows the comparison of the numerical analysis between the PhC-FBARs (triangular lattice PhC, as shown Figure 1a), SMRs, and Air-FBARs with the same piezoelectric stacks; it can be seen that the frequency of the PhC is lower than that of the Bragg-reflection-type SMR, but the impedance spectrum of the PhC-FBAR is smoother, and the spurious resonant peaks are also much lower than those of the SMR. However, as shown in Table 3, the resonant frequency and the quality factor of the PhC-FBAR are lower than those of the SMR. Theoretically, PhC is a kind of functional structure that exhibits a bandgap for acoustic or elastic waves, but it will not achieve a total reflection. For example, the effect of anomalous reflection will appear from PhC in some special conditions [43]. For the FBAR devices, the reflection property of PhC is a little worse than that of the SMRs. However, PhC has a greater attenuation rate in the bandgap. As a result, PhC-FBARs have far fewer spurious resonances and much smoother resonance curves, and their effective coupling coefficient is higher than that of SMRs.

**Figure 12:**
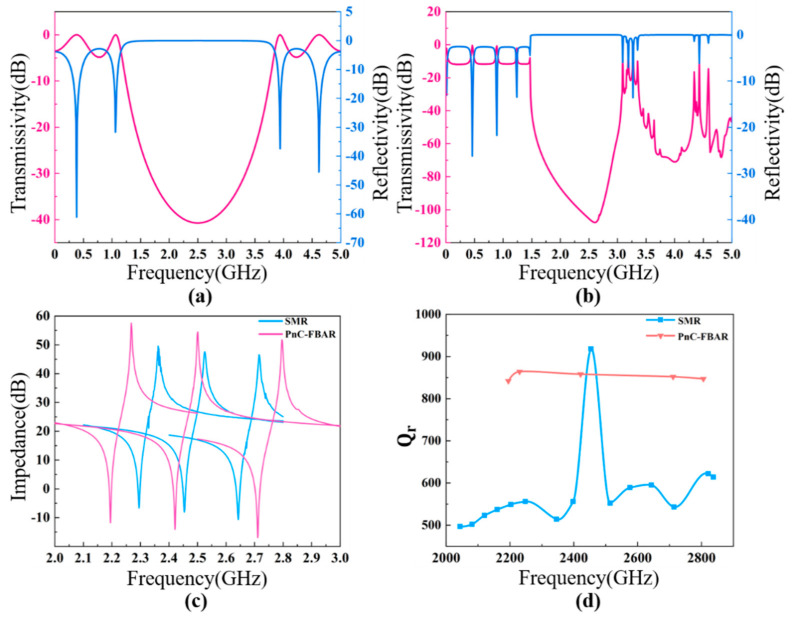
Transmissivity and reflectivity of the Bragg structure (**a**). Transmissivity and reflectivity of the square lattice PhC (**b**). Comparison of impedance curves at different working frequencies for SMRs and PhC-FBARs with the same acoustic isolation structure (**c**). Comparison of Q values at different working frequencies for SMRs and PhC-FBARs (**d**).

In some cases, especially for the filter application, the tuning of the working frequency of FBAR devices is inevitable. It would be tremendously useful to design an acoustic reflection layer that could work in a wide frequency range. To see whether the PhC-based acoustic reflection layers have this ability or not, we compared the frequency ranges that the PhC-FBAR and SMR could support with the same acoustic reflection structure. The Bragg layer used Mo as the high-acoustic-impedance material with a thickness of 625 nm, and ${\mathrm{SiO}}_{2}$ as the low-acoustic-impedance material with a thickness of 621 nm. Figure 12a shows the acoustic wave transmissivity and reflectivity of the SMR, showing that it has an optimal working frequency of ~2.5 GHz, and the reflectivity is zero dB for the frequency range from 1.7 GHz to 3.2 GHz. The transmissivity and reflectivity of the square lattice PhC are shown in Figure 12b, showing a frequency range from 1.7 GHz to 3.1 GHz with a unit reflection coefficient. To verify the acoustic energy isolation of the Bragg layers and the PhC, the thickness of the piezoelectric AlN film was varied to shift the resonant frequency of the devices. Impedances of the SMRs and PhC-FBARs with different frequencies are shown in Figure 12c, while the dependence of quality factor Q on frequency for the SMRs and PhC-FBARs is shown in Figure 12d. For the SMR, the Q decreases drastically when the working frequency is away from the central point, and the reduction in the Q values ranges from 32.2% to 46.5%, while the Q of the PhC-FBAR remains almost unchanged in the whole width of the frequency range investigated. In conclusion, PhC-FBARs could work at a much more comprehensive frequency range compared to SMRs when the same acoustic reflection structure is used.

## 5. Conclusions

In conclusion, this work introduces the basic theory of PhC and the calculation method of the band diagram of PhC. The band diagram and reflection coefficients of the four 2D PhC structures with square, square hollow, triangular, and honeycomb arrangements were calculated in the frequency range of 0–10 GHz using FEM analysis. Based on the reflection coefficients, the four structures of PhC-FBARs were studied and compared with those of the traditional Air-FBARs and SMRs. The results clearly show that the PhC has the ability to totally reflect the acoustic wave in the specific frequency range, and that the 2D PhC structures could provide wider bandgaps and richer bandgap characteristics. This paper provides a complete analysis of the structure of PhC-based FBAR devices, and their fabrication is the subject of ongoing research.

## Figures and Tables

**Table 1:** The material parameters of AlN and Mo.

Parameter			AlN	Mo
Density	ρ	$\left(\mathrm{kg}/m^{3}\right)$	3230	10,200
Elastic constant	c11	$\left(10^{9}N/m^{2}\right)$	345	-
	c12		125	-
	c13		120	-
	c33		395	-
	c44		118	-
Piezoelectric constants	e15		−0.48	-
	e31	$\left(C/m^{2}\right)$	−0.45	-
	e33		1.55	-
Relative permittivity	ε11	-	9	-
	ε33	-	11	-
Young’s Modulus	E	$\left(10^{9}N/m^{2}\right)$	-	312
Poisson’s ratio	μ	-	-	0.31

**Table 2:** Performance summary for four different structures of PhC-FBARs, and comparison with traditional Air-FBARs.

Structurer	$f_{r}\;(\mathrm{MHz})$	$f_{a}\;(\mathrm{MHz})$	∆f (MHz)	$K_{eff}\;\left(\%\right)$	$Q_{r}$	$Q_{a}$
Square lattice PhC	2421.3	2501.6	80.3	6.32	858.09	595.60
Air-FBAR	2465.6	2548.4	82.8	6.69	1065.62	1037.92
Square hollow cylinder lattice PhC	3380.2	3473.4	93.2	6.62	1012.89	933.57
Air-FBAR	3451.3	3550.1	98.8	6.68	1055.46	990.44
Honeycomb lattice PhC	2711.9	2797.6	85.7	6.58	931.96	850.90
Air-FBAR	2769.2	2858.1	88.9	6.84	1141.37	1055.74
Triangular lattice PhC	2438.4	2511.7	73.3	6.61	845.48	770.88
Air-FBAR	2465.6	2548.4	82.8	6.69	1065.62	1037.92

**Table 3:** Performance characteristics of FBARs with different structures.

Structurer	$f_{r}\;(\mathrm{MHz})$	$f_{a}\;(\mathrm{MHz})$	∆f (MHz)	$K_{eff}\;\left(\%\right)$	$Q_{r}$	$Q_{p}$
PhC-FBAR	2421.3	2501.6	80.3	6.32	858.09	595.60
Air-FBAR	2465.6	2548.4	82.8	6.69	1065.62	1037.92
SMR	2454.1	2525.4	71.3	5.54	918.09	802.08

## Data Availability

The data is available on reasonable request from the corresponding author.
